# The Role of Vaccination Interventions to Promote HPV Vaccine Uptake Rates in a College-Aged Population: a Systematic Review

**DOI:** 10.1007/s13187-020-01806-1

**Published:** 2020-06-21

**Authors:** Lihong Ou, Shawn D. Youngstedt

**Affiliations:** Edson College of Nursing and Health Innovation, Arizona State University, Phoenix, AZ USA

**Keywords:** Papillomavirus infection, Human papillomavirus vaccine, Vaccination promotion, Vaccination awareness, Immunization programs, Vaccination coverage, Young adults

## Abstract

This systematic review provided synthesized evidence regarding the effectiveness of the interventions promoting the human papillomavirus (HPV) vaccination in college-aged population. The HPV infection is the most prevailing sexually transmitted disease. Despite the availability and effectiveness of the 9-Valent HPV vaccine, the vaccine coverage among young adults remained low. In witness to the increasing burden of HPV-related infections and cancers, research focused on the vaccination interventions should be conducted to determine the effectiveness of the vaccination strategy and address the gap. The search was conducted through PubMed, Cochrane, and CINAL. Studies were included if they (1) included vaccination programs, (2) target population was young adults aged 17–26, (3) examined factors associated with the intervention effectiveness, (4) were published in English, and (5) were published between February 2010 and February 2020. HPV-related knowledge and intentions toward HPV vaccination were all reported increased after the intervention. Increased HPV vaccination intentions were found associated with the increased vaccine initiation and completion. Among bisexual or homosexual individuals, females were found more likely to complete the HPV dose 2 and 3. The review findings suggested using vaccination interventions incorporated with educational components to promote vaccine uptake among young adults. Supportive interventions tailored to different populations and settings are crucial to address the suboptimal HPV-related knowledge and vaccination status among the young beneficiaries.

## Background

Human papillomavirus (HPV) infection has been found as the most common sexually transmitted disease and greatly linked with genital infections such as genital warts. Specific types of HPV virus are also found responsible for cervical cancers, oropharyngeal cancers, and cancers of the penis, vagina, or anus [5]. Among which, 90 % of cervical and anal cancers can be attributed to the HPV infection, and around 70% oropharyngeal cancers caused by the virus [5].

Approximately over 70 million people in the USA were found infected with HPV sometime during the late adolescence and young adulthood, and fourteen million people are newly diagnosed with the infection each year [5]. Regarding the lifetime risk, most sexually active persons will have the HPV infection sometime in their lives, while most of them could not be aware of it [5]. Americans diagnosed with cancers caused by HPV infections are estimated around 27,000 every year [2].

The available 9-valent HPV vaccine is found effective in prevention of more than 90% HPV-related cancers [6]. The safety of the vaccine was also widely evaluated in clinical trials, and evidence were found in support of its safety and effectiveness in preventing infections and cancers caused by HPV [6]. The 9-valent HPV vaccine (Gardasil 9) was licensed in 2014 and recommended as the routine vaccination for HPV prevention by the Advisory Committee on Immunization Practices in 2015 [4].

Despite that the distribution of the 9-valent HPV vaccine started at the end of 2016, the vaccine uptake rates in the USA remained unsatisfactory and failed to meet the objectives of the Healthy People 2020 targeting for eliminating health disparities nationwide [4]. As per the vaccination report for 2014, the girls and boys who had completed the HPV 3-dose series were only 39.7% and 21.6%, respectively [7]. The vaccination gap warrants increasing efforts to disseminate information on HPV and its vaccine and improve the vaccine uptake rates to reduce the disease burden.

In review of the previous research, systematic reviews of HPV were primarily related to the prevalence and disparities in HPV infections, the efficacy and safety of the HPV vaccines, the measurement used in vaccine acceptability, the predictors or factors regarding HPV vaccine acceptability, and the role of health care providers in improving the vaccination initiation. The evidence they had compiled provided some insights into vaccination interventions aiming at improving the vaccination coverage, but the comprehensive review combining the factors which contribute to HPV vaccination, with vaccination intervention development and vaccine uptake evaluations, is scarce [1, 11, 13, 15, 17, 18]. One published review summarized 33 HPV educational interventions but without clearly identifying any superior interventions that could be widely recommended, and the inclusion of studies that are not addressing long-term assessment of vaccination rates might complicate its interpretation of success [12].

## Purpose

The aims of this study are to investigate the effectiveness of HPV vaccination interventions in college aged adults, critically evaluate and synthesize the intervention approach to improve vaccine coverage, also add insights into the feasibility and acceptability of the intervention, and further inform the practice and policy of the HPV vaccination among young adults. This present review embodies new emerging evidence and incorporates possible prerequisites for making informed decisions regarding HPV vaccination interventions at both the individual and community level. Countries out of the USA which achieved promising results of HPV vaccination campaigns were also included in the review to provide more insights across nations.

## Methods

### Search and Screening Strategy

This author searched the PubMed, Cochrane, and online version of Cumulative Index to Nursing & Allied Health Literature (CINAL) independently to identify peer-reviewed studies published between February 2010 and February 2020. The search was limited to English language and with keywords and relevant Medical Subject Headings (MeSH) subject terms, including HPV, human papillomavirus, vaccine or vaccination, immunization, uptake, intervention, and students. Finally, references from systematic reviews and references from the studies identified were also assessed for inclusion in the review process.

Studies were eligible for inclusion if the following criteria were fulfilled: interventions were developed to increase HPV-related knowledge and vaccination rate and provided quantitative data for HPV vaccination coverage specifically. Interventional, observational, and systematic studies were all eligible. Studies that only provided qualitative results, only published as abstracts and protocols, or only assessed HPV knowledge, awareness, attitudes, intentions, and barriers to vaccination were excluded. Preferred Reporting Items for Systematic Reviews and Meta-Analyses (PRISMA) recommendations were followed during the search, both article titles and abstracts were scrutinized first, and then relevant full-text articles were obtained and reviewed by this author to further confirm that verification criteria were met.

### Data Extraction and Analysis

This author extracted information about intervention and assessed risk of bias. The risk of bias and the certainty in evidence were evaluated for each study by using the Cochrane Grading of Recommendations Assessment, Development and Evaluation (GRADE) approach. This approach results in three level of risk of bias and four levels of quality of evidence. Critical factors for determining the grade for each study are listed in detail for the approach, including the risk of bias, evidence quality rating scale, and descriptions of the ratings.

## Results

### Sample

Seventy-eight references had been screened, and a total of 10 relevant articles met the selection criteria for this review. One (10.0%) of the studies was a cross-sectional analysis [8]. Three (30.0%) were quasi-experimental designs [14, 19, 21], and six (60.0%) were randomized controlled trials [3, 10, 16, 20, 22, 23] (see Appendix Fig. 1 for the flowchart of included and excluded studies).

### Demographic Characteristics

The study participants’ ages ranged from 18 to 38 years old. The majority of the studies (*n* = 9) focused on college students, and one study included both employed women and undergraduate students for comparison. Three studies were conducted outside of the USA, including one in China and two in Canada. Sample size of participants in the studies ranged from 200 to 1703. Four studies recruited both male and female participants, with six studies focused on females only (see Table 1 for characteristics of the selected studies).

**Table 1:** Characteristics of the selected studies (*n* = 10)

Characteristic	Number of studies	%
Geographic origin		
North America	7	70.0
Canada	2	20.0
China	1	10.0
Gender		
Females	6	60.0
Females and males	4	40.0
Study design		
Cross-sectional analysis	1	10.0
Quasi-experimental designs	3	30.0
Randomized controlled trials	6	60.0
Sample size		
200–400	7	70.0
401–600	1	10.0
601–1000	1	10.0
1001–1200	1	10.0
Intervention setting		
Lecture	1	10.0
Electronic intervention	3	30.0
Campus-based/student health clinic	2	20.0
Mixed	4	40.0
Theoretical framework guided		
Yes	5	50.0
No	5	50.0

### Intervention Characteristics

All the interventions targeted for boosting the human papillomavirus (HPV) vaccine acceptability and coverage among young adults. Among the ten interventions reviewed, five studies described the use of theory-based interventions, and key factors of the Health Belief Model (HBM), the Transtheoretical Model (TTM), and the Information, Motivation, Behavioral skills model (IMB) were reported to be applied to improve design of the interventions [14, 16, 19, 20, 23]. Critical elements based on core assumptions of the HBM and the Theory of Planned Behavior (TPB) were also incorporated into the development of the instruments to evaluate the benefits of interventions [20, 23]. The tailored interventions moved beyond general interventions by incorporating educational, cognitive, and behavior change components and following up reminders. Topic web pages, educational videos, computer-tailored interventions, and electronic text messages were used as various modalities in support of conveying the health education messages.

### Data Collection and Measurement

A range of pre- to post-intervention survey questionnaires and human papillomavirus (HPV) vaccine initiation and completion rates were used to evaluate the effectiveness of the interventions. Reasons stated for being unvaccinated were explored as well. The follow-up evaluations were conducted 3 months up to 1 year to measure the HPV vaccine dose initiation and completion. Instruments used in the studies examined the topics covering HPV-related and HPV vaccine knowledge and awareness, HPV and HPV vaccine beliefs and decision-making factors, self-efficacy, and vaccine uptake. Most of the studies used self-reported measurements, and two studies (20%) reviewed medical records for vaccine uptake assessment [20, 23]. One study also addressed the appraisal of the intervention by examining its acceptability and feasibility [19]. Nine of the ten studies adopted the questionnaires without identifying their validity, in particular, four studies reported internal consistency of the instrument (Cronbach’s *α* = .75~.95) [14, 16, 19, 21]. The description of the psychometric properties of instruments used in two studies was missing [3, 10].

### Critical Appraisal

All the studies lacked in-depth description of participant attritions, most of the studies (*n* = 7) did not address adequately follow-up, and nine studies had flawed measurement of outcomes due to the limited evidence of the instrument validity. Two studies were found to be of low risk of bias, four of medium risk, and four of high risk. Seven of the ten studies included in the review were graded as moderate level of quality of evidence, as a result of the majority of randomized controlled studies(*n* = 4) not specifying allocation concealment and blinding procedures, one study being observational and flawed design and measurements as potential threats to construct, internal, and external validity. Two randomized controlled studies discussed randomization procedures, one described the use of allocation concealment mechanism with sequentially numbered, opaque sealed envelopes, and another described that the investigators and research nurses in the field were blinded to the allocation sequence [20, 23] (see Table 2 for quality rankings of studies in the review).

**Table 2:** Quality ranking of studies using the GRADE criteria (Grading of Recommendations Assessment, Development and Evaluation)

Studies	GRADE quality of evidence
Piedimonte et al. (2018)	Moderate
Bennett et al. (2015)	Moderate
Richman, Maddy, Torres, and Goldberg (2016)	Moderate
Cory et al. (2019)	Moderate
Chang et al. (2013)	Low
Krawczyk et al. (2012)	Moderate
Paiva, Lipschitz, Fernandez, Redding, and Prochaska (2014)	Moderate
Hayes, Pan, Kunkel, Mcgivney, and Thorpe (2019)	Moderate
Patel et al. (2012)	High
Vanderpool et al. (2013)	High

### Effects of Interventions

Prior to the vaccination interventions, the level of baseline HPV-related knowledge and intentions toward HPV vaccine uptake was generally identified low, and the knowledge and vaccine beliefs were found increased after interventions. Despite the significant increase in knowledge, no associations were identified between the knowledge increase, risk perception, and intention to be vaccinated. Theoretically driven interventions were found as more effective strategies for increasing the vaccine intentions and uptake, compared with those who were not guided by behavioral change theories. Individual beliefs about vaccinations and vaccination intentions were defined as the precursor to the vaccine behavior changes. Gender was also found as the biggest predictor of HPV vaccine completion, with 2.35 times more likely to complete HPV series among female students, and higher vaccine completion rates were also seen in participants who were self-identified as homosexual or bisexual [22].

Among the vaccination programs using intervention theories, participants enrolling in the HBM-embedded interventions demonstrated low perceived vaccination barriers and high perceived benefits of the vaccination [14, 16]. For interventions developed under the influence of the TPB and the IMB constructs, successful outcomes were identified as 43.3% participants’ completion of the 3-dose vaccine series after the intervention, which was 2.44 times more than that of the usual care group; in addition, participants who demonstrated vaccine intentions were found ten times more likely to initiate the vaccination than those without the intentions [20, 23]. Favorable outcomes related to enhanced health beliefs and intentions to vaccinate were also found in educational sessions and campaigns facilitated by medical, pharmacy students, health clinic residents, and social media [14, 21].

## Discussion

The systematic review includes 10 studies in total and investigated the effectiveness of the vaccination interventions in improving the awareness of the human papillomavirus (HPV)-related infections, HPV vaccination intentions, and the vaccine uptake rates among college students aged 17–26. Constructs of the interventions and evaluations of the outcomes were also identified.

In the review, all studies included vaccination interventions or programs that promoted the vaccination intentions and behaviors among college-aged students. Most of the studies assessed HPV-related knowledge, including the knowledge of cervical cancer, individuals’ perception of the risk of the disease, attitudes or intentions toward vaccination, and perceived barriers to the vaccination. The study participants’ intentions of receipt of the vaccine were found positively linked with improved awareness and knowledge of HPV-associated infections. These results imply that emphasizing the educational components in the development of vaccination intervention would improve the effectiveness of the intervention.

A significant increase in the HPV-related knowledge, attitudes, and acceptability of the HPV vaccine were found in the study participants upon completion of the interventions [10, 14, 16, 19–22]. However, in general, a deficit of knowledge on HPV infection and the associated diseases was identified in pre-intervention period in the systematic review. Most of the study participants presented an absence of HPV education or lack of the knowledge about HPV risk factors, mode of transmission, symptoms, HPV-related cancers, genital warts, and prevention of HPV. Non-White participants demonstrated significantly lower scores on the HPV-related knowledge scale, compared with the White group [19]. Female participants were found as the largest predictor of the completion of the vaccine series [22]. These findings might be attributed to the misconception that females are the only potential victims of HPV-related diseases and the effectiveness of HPV vaccine is for preventing cervical cancer alone. Increasing the awareness that both males and females could be infected with the virus and suffer from the complications of HPV infections and the dissemination of HPV-related knowledge among men is critical for reducing the risk of HPV-caused infections. The number of participants identifying as bisexual or homosexual that completed the HPV dose 2 was more than that of the participants identifying as heterosexual [22]. This is in contrast to another study conducted in Chinese young men, in which the authors identified that the risk of HPV infection was higher and the HPV vaccine uptake rates were lower in bisexual-/homosexual-identified participants [9]. These findings would highlight the needs of tailored interventions to address the gender disparity and the difference in sexual orientation and ethnic groups to overcome the inequality in the HPV vaccination.

Additionally, perceived severity of HPV infection, susceptibility to the HPV infection, and benefits of the vaccine were identified among the factors that positively affected the participants’ intentions to get vaccinated [14, 16]. Addressing the key components that contribute to vaccination behavior intentions such as perception of the infection risks and benefits of the vaccine could possibly lead to higher acceptability of the vaccination. In particular, possible exposures to HPV infections were found not necessarily associated with increased likelihood of taking the vaccine. Although the ones who are infected with HPV could still benefit from the HPV vaccine, and the prevalence was found high in sexually active persons, participants who were sexually active reported less likely to intend to have the vaccination, compared with participants who were not currently sexually active. The authors discussed that this might be due to their concerns of the effectiveness of the vaccine, and the participants could possibly consider that it was too late for them to have the vaccine [20]. The response addresses the needs for narrowing the knowledge gap in the HPV vaccine which might refrain people from getting the timely protection.

Two major types of interventions emerged in the review, those with the theory-based approaches and those without. Promising health care outcomes were identified in both types in terms of the increased level of knowledge about the HPV infections and HPV vaccine. Increase in the HPV knowledge was found in the immediate post-intervention surveys while with one exceptional study identified significant improvement in HPV knowledge 3 months following the intervention [3]. Further exploring of the influential factors contributing to the knowledge increase might be needed. Nevertheless, this phenomenon could possibly be explained by the carryover effect, as the same question survey was used to collect the data for the two time points (the baseline survey and the 3-month survey), participants’ responses on a second administration could be influence by their memory of initial responses. Interventions that incorporated with cognitive or behavior change constructs generated more vaccination behaviors as a consequence of increased vaccination intentions and beliefs, increased perceptions of the vaccine benefits, and low perceived barriers to vaccination. However, only three of the studies included up to a 6-month follow-up period [21–23]. The 6-month follow-up evaluation of vaccination rates would be a definitive sign of intervention success as the 3-dose schedule of the HPV vaccine is recommended for individuals who initiates the vaccine at the age of 15 through 45 and would be completed at 0, 1, or 2 months and 6 months, respectively. Apart from the aspects of advancing knowledge, increasing personal sense of control over actual vaccination behavior is also pivotal to vaccination intervention success.

The vaccination interventions identified in the review were also varied in settings and modalities to deliver. Most of the interventions had been completed in a one-time-only session, and reinforcement afterwards was provided in the form of reminding messages for the vaccine appointment or providing additional educational information [20–22]. The education reinforcement activities might have an impact on the change of the follow-up scores in the knowledge assessment, and participants’ attitudes and knowledge toward the HPV vaccine could be modified by their learning experience between short-term and long-term assessments. There is a lack of information specifying which elements in the intervention attributed to favorable outcomes among participants in view of the variety of online-based educational interventions and school-based clinical visits available in the review. Establishing a supportive learning environment and catering to participants’ needs in the development of educational interventions could facilitate participants’ engagement and yield more effective outcomes. Interprofessional collaborative efforts could also empower the intervention to reduce the disparities in vaccinations. Incorporating use of resources like interactive slideshows, social media, and advertising consultation groups in the vaccination campaign showed positive results in participants’ attitudes and their willingness to be vaccinated [21]. Integrating a variety of avenues of health communication as powerful tools could be beneficial to address the vaccination gap. The researchers also discussed the campaign initiatives that having onsite vaccination immediately after the intervention, this highlights the feasibility and importance of completing the vaccination on the same day of the intervention. Part of the reason might be due to peer stereotyping and pressure; participants’ perceptions of risks and vaccination needs could be influenced by subtle cues such as sensing others’ vaccination intentions and behaviors at the scene. Subjective norms or social norms could further refrain participants from being vaccinated; nevertheless, maternal and peer influences were found not associated with vaccination initiation [23]. Further studies are necessary with respect to explaining how parental or peer influence on individual’s HPV vaccination behaviors would be like during early adulthood, as well as exploring the difference of the influences on vaccination initiation and completion. On the other hand, the availability of vaccination reimbursement could affect participants’ decisions on taking the vaccine; these findings might warrant further studies on the vaccination gap in the underinsured or the unemployed population. Financial concerns of the vaccine could be a key barrier to vaccine acceptance.

This review only included peer-reviewed articles to facilitate the inclusion of relatively good quality work. During the review, a systematic approach was followed based on PRISMA checklist, and critical appraisal of each article was conducted following the guidance of the GRADE approach. The overall quality of the included studies was moderate, and campus-based convenience sampling, insufficient description of participant attritions and allocation concealment and blinding procedures in trial studies, self-reporting measures, and limited evidence of the instrument reliability and validity were found as the major factors that could possibly increase the risk of bias in the studies and jeopardize the transferability and generalizability of the study results. Several areas were prompted for future studies, which primarily centered on long-term assessments of the intervention effectiveness; development and evaluations of tailored vaccination interventions to manage the gender disparity, socioeconomic disparity, diversity in sexual orientation, and different ethnic groups; the difference in parental, peer, and interprofessional roles; and impact in health prevention regarding HPV-related infections and cancers. Moreover, in the light of generally low level of knowledge of HPV prevention and better immune response with the younger population, effective strategies addressing comprehensive and quality sexual health education at younger ages should be examined.

## Conclusion

On the whole, this review indicated the complex of contributing factors to a successful HPV vaccination intervention. The approaches adopted in the studies achieved higher level of HPV-related knowledge, vaccination intentions, and higher vaccination rates in comparison of the pre-intervention status. Insights and gaps identified in the review could possibly be applied to guide sexual health education and inform the policy of the HPV vaccination and other vaccine preventable diseases, especially for the vaccinations that are not included in the national program. More robust study designs for studying the associations between HPV vaccination interventions and vaccine uptake rates across different settings and populations are also underlined in the findings of the review.

## Figures and Tables

**Fig. 1:**
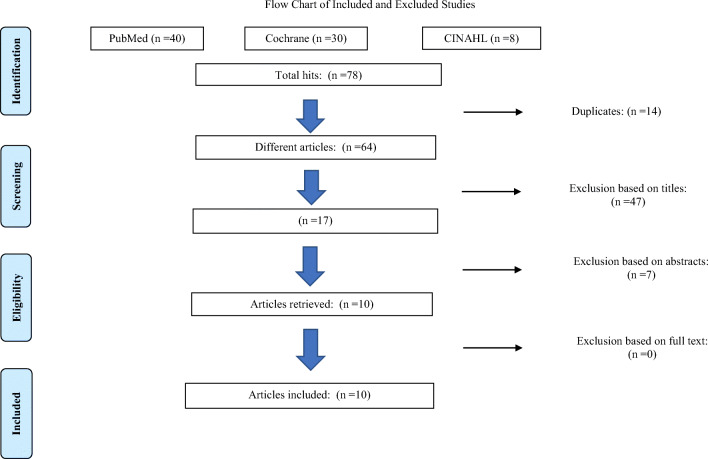
Flowchart of included and excluded studies

## Data Availability

The findings of the study are available from the corresponding author upon reasonable request.
